# PET/CT deep learning prognosis for treatment decision support in esophageal squamous cell carcinoma

**DOI:** 10.1186/s13244-024-01737-1

**Published:** 2024-06-24

**Authors:** Jiangdian Song, Jie Zhang, Guichao Liu, Zhexu Guo, Hongxian Liao, Wenhui Feng, Wenxiang Lin, Lei Li, Yi Zhang, Yuxiang Yang, Bin Liu, Ruibang Luo, Hao Chen, Siyun Wang, Jian-Hua Liu

**Affiliations:** 1 School of Health Management, China Medical University, Shenyang, China; 2 Department of Medical Imaging, Zhuhai People’s Hospital (Zhuhai Clinical Medical College of Jinan University), Zhuhai, China; 3 The Fifth Affiliated Hospital of Sun Yat-Sen University, Guangdong, China; 4 Department of Surgical Oncology and General Surgery, The First Hospital of China Medical University, Key Laboratory of Precision Diagnosis and Treatment of Gastrointestinal Tumors China Medical University, Ministry of Education, Shenyang, China; 5 Department of VIP In-Patient Ward, The First Hospital of China Medical University, Shenyang, China; 6 Department of Radiology, The Second People’s Hospital of Xiangzhou, Zhuhai, China; 7 Department of Radiology, Guangdong Provincial People’s Hospital (Guangdong Academy of Medical Sciences), Southern Medical University, Guangzhou, China; 8 Department of Computer Science, The University of Hong Kong, Hong Kong, Hong Kong; 9 Department of Gastroenterology, Guangdong Provincial People’s Hospital, Guangdong Academy of Medical Sciences, Southern Medical University, Guangzhou, China; 10 Department of Oncology, Cancer Center, Guangdong Provincial People’s Hospital (Guangdong Academy of Medical Sciences), Southern Medical University, Guangzhou, China

**Keywords:** Esophageal cancer, Deep learning, Adjuvant chemotherapy, PET-CT, Prognosis

## Abstract

**Objectives:**

The clinical decision-making regarding choosing surgery alone (SA) or surgery followed by postoperative adjuvant chemotherapy (SPOCT) in esophageal squamous cell carcinoma (ESCC) remains controversial. We aim to propose a pre-therapy PET/CT image-based deep learning approach to improve the survival benefit and clinical management of ESCC patients.

**Methods:**

This retrospective multicenter study included 837 ESCC patients from three institutions. Prognostic biomarkers integrating six networks were developed to build an ESCC prognosis (ESCCPro) model and predict the survival probability of ESCC patients treated with SA and SPOCT. Patients who did not undergo surgical resection were in a control group. Overall survival (OS) was the primary end-point event. The expected improvement in survival prognosis with the application of ESCCPro to assign treatment protocols was estimated by comparing the survival of patients in each subgroup. Seven clinicians with varying experience evaluated how ESCCPro performed in assisting clinical decision-making.

**Results:**

In this retrospective multicenter study, patients receiving SA had a median OS 9.2 months longer than controls. No significant differences in survival were found between SA patients with predicted poor outcomes and the controls (*p* > 0.05). It was estimated that if ESCCPro was used to determine SA and SPOCT eligibility, the median OS in the ESCCPro-recommended SA group and SPOCT group would have been 15.3 months and 24.9 months longer, respectively. In addition, ESCCPro also significantly improved prognosis accuracy, certainty, and the efficiency of clinical experts.

**Conclusion:**

ESCCPro assistance improved the survival benefit of ESCC patients and the clinical decision-making among the two treatment approaches.

**Critical relevance statement:**

The ESCCPro model for treatment decision-making is promising to improve overall survival in ESCC patients undergoing surgical resection and patients undergoing surgery followed by postoperative adjuvant chemotherapy.

**Key Points:**

- ESCC is associated with a poor prognosis and unclear ideal treatments.
- ESCCPro predicts the survival of patients with ESCC and the expected benefit from SA.
- ESCCPro improves clinicians’ stratification of patients’ prognoses.

**Graphical Abstract:**

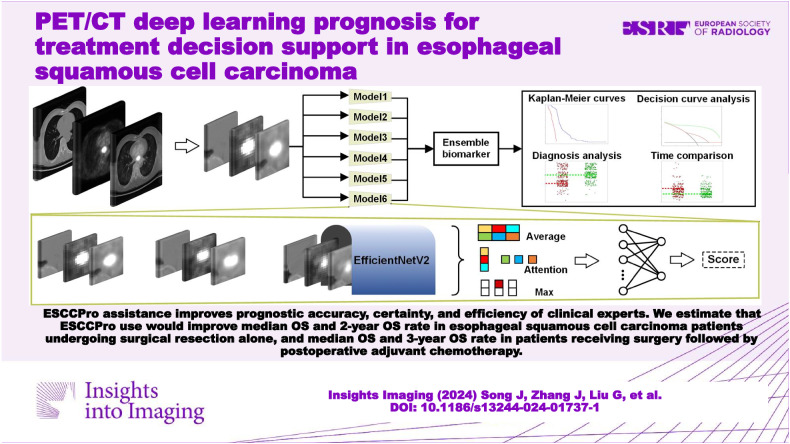

## Introduction

Esophageal cancer is the sixth leading cause of cancer mortality worldwide [1]. Esophageal squamous cell carcinomas (ESCC) are malignant tumors originating from esophageal squamous epithelium mucosal cells. They have varied prognoses and are the most common esophageal cancer subtype, with a 5-year survival of 15–20% in patients with locally advanced ESCC [2, 3]. Although surgery followed by postoperative adjuvant chemotherapy (SPOCT) can improve the survival of these patients, its use remains controversial [4]. Clinical studies have shown that only half of the treated patients obtain complete/partial pathologic responses with SPOCT [3, 5–8], therefore, controversy over implementation criteria exists [9, 10].

Clinical decision support is critically important for SPOCT as clinical responses and long-term survival prognosis vary substantially in patients with ESCC [11]. Despite imaging diagnosis and pathological staging contributing to SPOCT precision, high levels of diagnostic expertise are often scarce. Therefore, developing accurate non-invasive prognostic models could help define subgroups of patients who would benefit from SPOCT and identify patients who might receive a better cost-to-benefit ratio from surgery without SPOCT owing to non-extendable survival outcomes.

One potential solution could be to adopt artificial intelligence (AI) in the clinical management of patients with ESCC. Recently, AI has been used in medical image analysis and has performed well in several diagnostic, prognostic, and predictive tasks [12–15]. Of these, computed tomography (CT)-based AI models have been developed for treatment response prediction and survival prognosis of adjuvant chemotherapy in patients with ESCC [16, 17], and F-18 fluorodeoxyglucose positron emission tomography (PET)-based radiomics models have been proposed in survival benefit evaluation of chemoradiation [18–20]. Although progress has been achieved, there remains uncertainty regarding SPOCT benefits, and patients who probability cannot obtain expected survival outcomes from the treatment should be identified beforehand to avoid additional treatment burden and clinical risk [10]. However, previous studies have not investigated the appropriate criteria to manage this issue.

This study aimed at proposing a pre-therapy PET/CT image-based deep learning approach for ESCC prognosis (ESCCPro). The objective of ESCCPro is to identify the patients with ESCC who are more suitable for surgical resection alone (SA) and the patients with ESCC who could obtain better survival benefits from SPOCT. In addition, the ESCCPro is designed to improve the prognostic accuracy, certainty, and efficiency of clinical experts in the clinical treatment decision-making in ESCC.

## Methods

### Patients

**Fig. 1:**
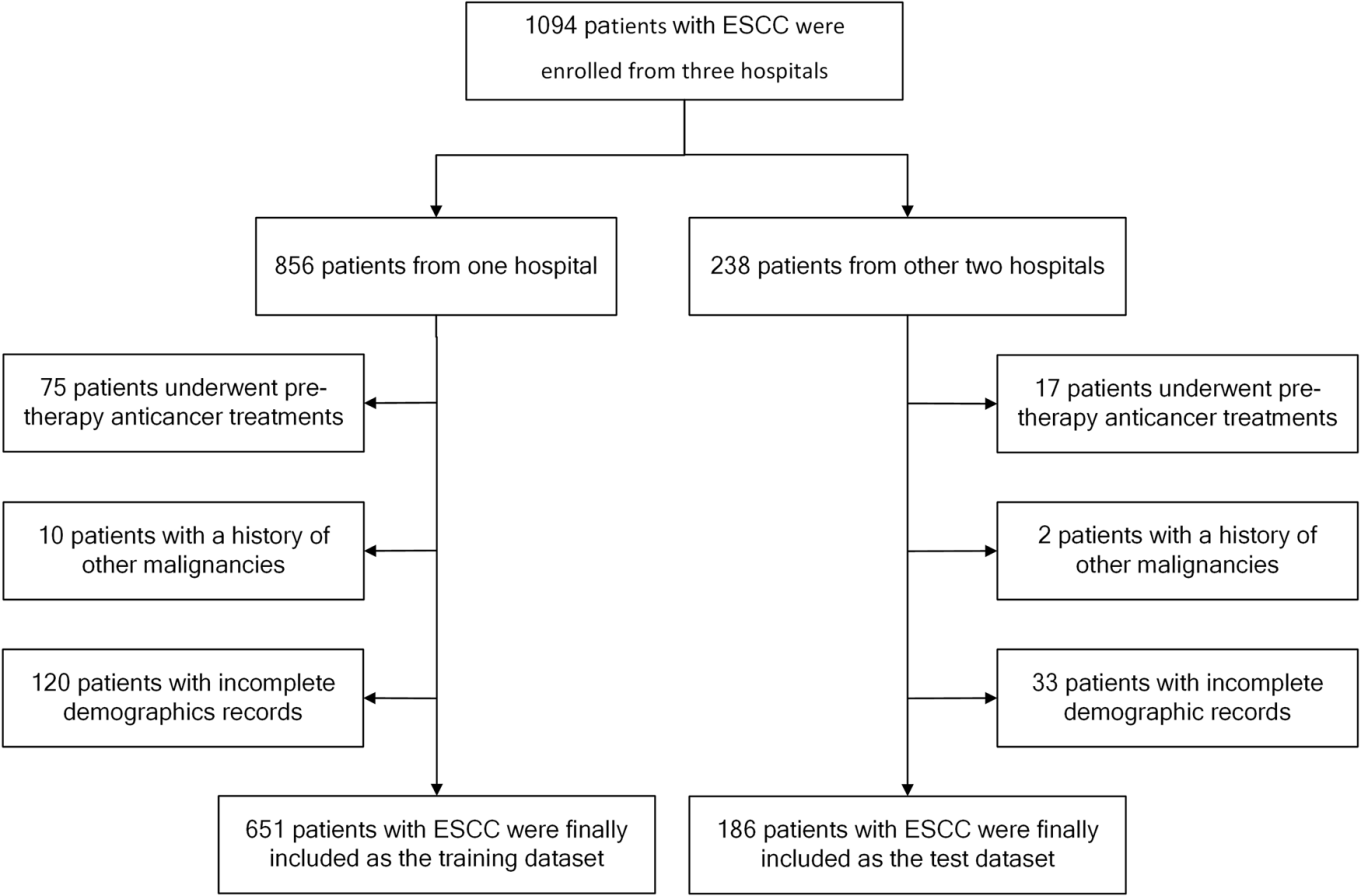
Patient enrollment in this study

Three independent hospitals were included. The training dataset consisted of patients enrolled from Guangdong Provincial People’s Hospital between January 01, 2012 and December 31, 2019. The test dataset consisted of patients from two other hospitals (Zhuhai People’s Hospital and The Fifth Affiliated Hospital of Sun Yat-Sen University) enrolled between January 01, 2017 and December 31, 2021. A flowchart of patient inclusion and exclusion is shown in Fig. 1, and details are presented in Supplementary A3.

Both surgery alone (SA) patients and surgery followed by POCT patients (SPOCT) were included at the T1bN0M0–T3N1M0 stage. In addition, due to the limited physical tolerance, patients with advanced-stage ESCC are potentially unable to receive surgery or chemotherapy. These patients were included in this study as a control cohort (details see Supplementary A3).

Regular follow-up (3–6 months) was performed in accordance with the previous ESCC studies [21–23]. For long-term survival evaluation, OS, defined as the interval from treatment initiation until death or last observation, was used as the primary endpoint. For treatment response evaluation, disease-free survival (DFS) was used as the secondary endpoint for patients who underwent SA/SPOCT and was measured from the date of surgery to that of disease recurrence or metastasis onset. For the control cohort, progression-free survival (PFS) was used as the secondary endpoint, defined as the interval from the date of initial treatment to disease progression. Patients without the observed endpoints, those lost to follow-up, or those alive at the last follow-up were censored.

### ESCCPro building and validation

All enrolled patients underwent pretreatment [^18^F]FDG PET/CT scan. The details for PET/CT acquisition and manual segmentation of all primary ESCC tumors are presented in Supplementary A4. Additionally, 100 patients were randomly selected for manual segmentation by another radiologist to evaluate inter-observer bias. After a five-month washout period, the radiologist was requested to re-segment the 100 patients using the same software to evaluate the intra-observer reproducibility.

**Fig. 2:**
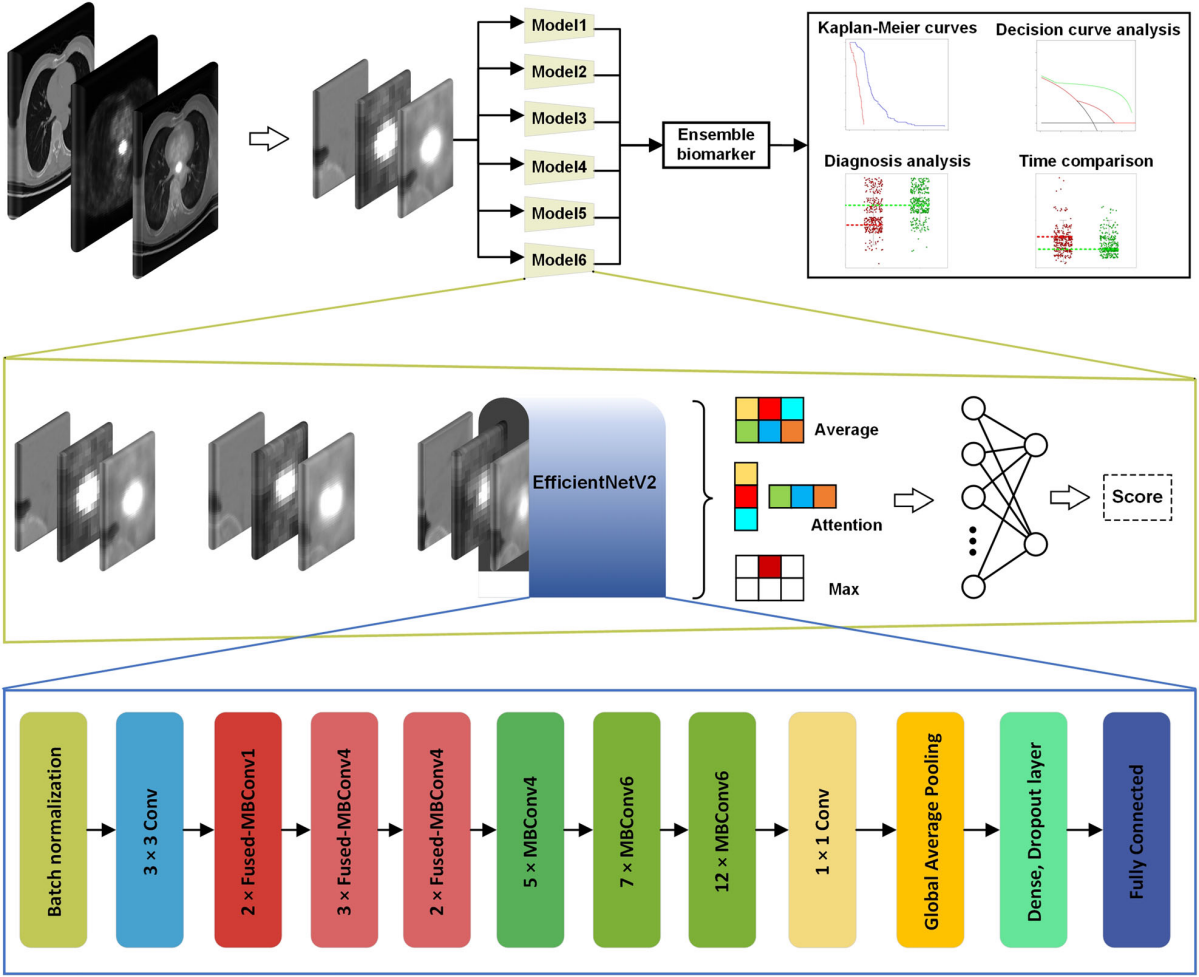
The flowchart of the proposed ESCCPro model. A total of six EfficientNetV2-derived networks were adopted for the ensemble biomarker, and each of them comprised of a representation network (EfficientNetV2), a pool function (averaged pooling, adaptive attention pooling, or max pooling), and a fully connected classification network. The ensemble biomarker was based on a Cox regression of the output of the six models, and the entire network was trained end-to-end

Six networks were trained using the training dataset. All networks were derived from the latest EfficientNetV2 network, a proven network for cancer detection and prognosis [24–26], which we designed to classify PET/CT tumor images. The ESCCPro model was built based on the output of the six models, and each model was comprised of an EfficientNetV2 representation network, pooling function, and fully connected classification, as shown in Fig. 2. PyTorch (version 1.7.1) was used for model training. After the network loss of ESCCPro on the training dataset reached a stable state with no further decrease, the model was locked and validated on the testing dataset. The details of the ESCC, including the EfficientNetV2 and the pooling functions and hyperparameters of the six networks are listed in Supplementary A2. The entire network was trained end-to-end (using image input directly to predict patient outcomes, Fig. 2), and each training iteration used a batch size of 16 images. The ensemble ESCCPro prognostic signature was constructed using a Cox regression of the output of the six networks (Fig. 2).

Based on the previous studies, the median overall survival (OS) of patients with ESCC who underwent SA was approximately 18–27 months (18.4 months in this study), and that of patients who received SPOCT was approximately 27–33 months (30.3 months in this study) [27, 28]. To accurately distinguish patients with distinct survival prognoses, SA patients were first divided into SA-good and SA-poor prognostic subgroups based on the median OS. The SPOCT patients were then divided into SPOCT-good and SPOCT-poor subgroups using the same criteria. An ESCCPro-SA model was first used to evaluate the probability of SA-good or SA-poor outcomes. Subsequently, an ESCCPro-SPOCT model was built to suggest the potential benefits of POCT after surgery. This design aligned with the actual clinical workflow for developing treatment regimens for patients with ESCC. The two models were trained and validated with OS as the primary endpoint for long-term survival prognosis, verified using DFS (secondary endpoint) for post-treatment response evaluation, and finally validated on the test dataset. The control cohort was used for comparison with the ESCCPro-predicted subgroups.

### Clinical assistance evaluation

Seven clinical experts from radiology, nuclear medicine, surgery, and oncology, with diverse levels of experience in ESCC treatment (2–15 years), evaluated the prognosis of 50 SA and 50 SPOCT patients who were randomly selected (10% from training and 90% from test data). The first half of the group (25 SA and 25 SPOCT patients) was presented to clinicians with demographic and baseline clinical information. In addition to the above information, the last half of the group was presented with the ESCCPro prediction score and accuracy. The time per patient was recorded, and diagnosis confidence was recorded (very certain, certain, neither, uncertain, and very uncertain).

### Statistics

Differences in the clinical characteristics between datasets were calculated using Fisher’s exact tests or chi-square tests for categorical data and Wilcoxon signed-rank tests for continuous variables. A detailed statistical analysis of this study has been presented in Supplementary A3. Two-sided *p* values of < 0.05 were regarded as statistically significant and all analyses were conducted with R version 3.4.3.

## Results

### Patients

A total of 651 patients with ESCC were eligible for the training dataset (52 censored data), including 219 SA patients (171 men), 174 SPOCT patients (156 men), and 258 patients in the control cohort (201 men). Additionally, 186 patients were enrolled in the test dataset (20 censored data), including 62 SA patients (50 men), 57 SPOCT patients (49 men), and 67 patients in the control cohort (51 men).

The median OS (with [inter-quartile range]) across the datasets were 18.4 [11.2, 29.4] months, 30.3 [16.1, 65.6] months, and 9.2 [5.0, 20.3] months for SA patients, SPOCT patients, and control group patients, respectively. No significant difference was found in the OS of the patients in the SA, SPOCT, and control cohorts between the training and test datasets (*p* > 0.05). The median OS of the SA and SPOCT groups were used to define subgroups with good or poor survival outcomes. For the DFS of SA patients, a significant difference was found between the two datasets (*p* = 0.021). For the SPOCT and control cohorts, no significant difference was found in the DFS between the training and test datasets (*p* > 0.05, Table 1).

**Table 1:** Characteristics of the included patients

	Training dataset	Test dataset	*p* value
Number	651	186	0.637
SA (%)	219 (33.6)	62 (33.3)	
SPOCT (%)	174 (26.7)	57 (30.6)	
Control (%)	258 (39.7)	67 (36.1)	
Age, years (SD)	61.5 (9.3)	62.7 (10.5)	0.900
Sex (%)			0.826
Male	528 (81.1)	150 (80.6)	
Female	123 (18.9)	36 (19.4)	
Primary tumor size (cm)	5.1	6.8	0.178
Surgery (%)			0.330
Radical surgery	201 (91.8)	55 (88.7)	
Palliative surgery	18 (8.2)	7 (11.3)	
Chemotherapy (%)			0.861
Taxane + Platinum	167 (96.0)	53 (92.9)	
Paclitaxel + Cisplatin	7 (4.0)	4 (7.1)	
Median OS			
SA (months, SD)	18.9 (19.1)	16.8 (15.3)	0.558
SPOCT (months, SD)	30.5 (35.5)	28.1 (29.1)	0.329
Control (months, SD)	10.1 (9.0)	7.3 (7.5)	0.891
Median DFS/PFS (SD)			
SA (months, SD)	16.9 (13.3)	9.0 (10.5)	0.021*
SPOCT (months, SD)	16.3 (12.5)	11.5 (9.9)	0.098
Control (months, SD)	6.0 (6.2)	5.0 (4.5)	0.771

*p* values indicate the differences in the proportion of data between the two datasets (*indicates statistically significant difference)

*SA* surgery alone, *SPOCT* surgery followed by postoperative adjuvant chemotherapy, *Control* the patients in the control cohort, *SD* standard deviation

### ESCCPro building and validation

**Fig. 3:**
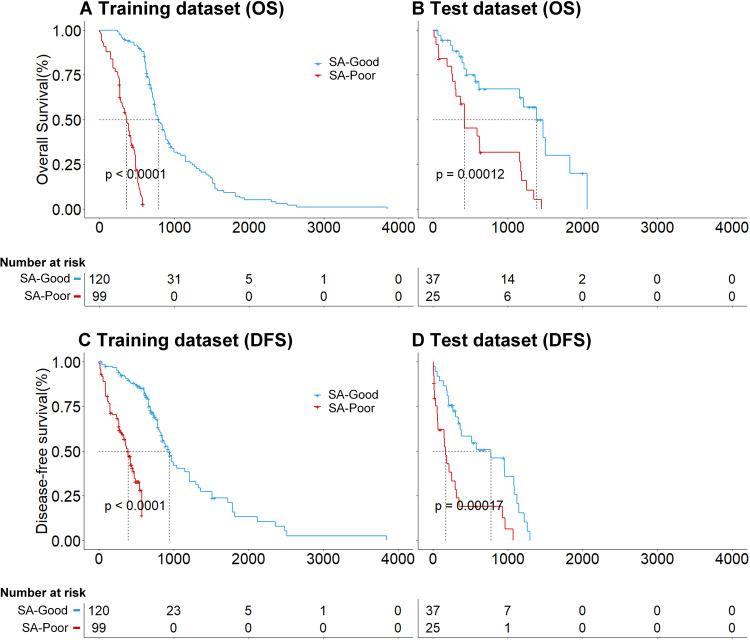
Kaplan–Meier survival curves of the ESCCPro-SA predicted good and poor outcomes for patients treated with SA. Significant differences were found for both OS (**A**, **B**) and DFS (**C**, **D**) prognosis for the training and test datasets (*p* < 0.05)

**Fig. 4:**
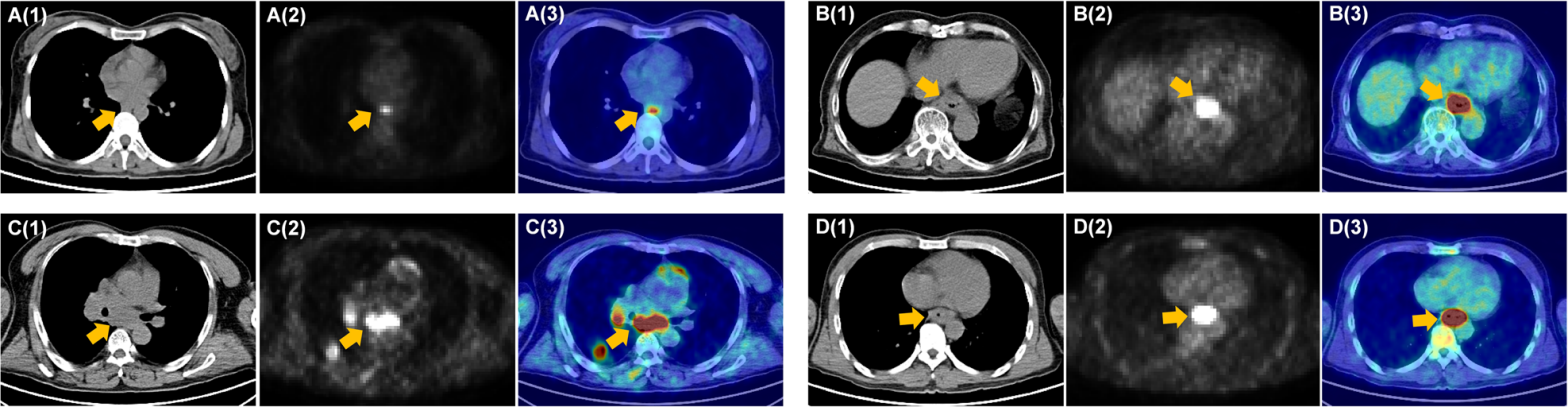
CT, PET, and PET/CT fusion images of two patients who underwent SA: **A** A 38-year-old woman with low-differentiated squamous cell carcinoma in the middle of the chest (ESCCPro-SA score: 0.11, OS: 26.6 months). **B** A 74-year-old man with low-differentiated squamous cell carcinoma in the lower thoracic segment (ESCCPro-SA score: 0.95, OS: 3.0 months). Two patients underwent surgical resection plus postoperative adjuvant chemotherapy: **C** A 68-year-old man with moderate-differentiated squamous cell carcinoma in the middle of the chest (ESCCPro-SPOCT score: 0.09, OS: 100.0 months). **D** A 51-year-old man with moderate-differentiated squamous cell carcinoma in the lower thoracic segment (ESCCPro-SPOCT score: 0.75, OS: 15.1 months)

In an actual clinical scenario, the decision of surgery resection in patients with ESCC was the first task of this study. For ESCCPro-SA, an optimal cut-off of 0.8 was determined by X-tile, and the patients were classified as SA-poor (high scores) or SA-good (low scores). The predicted median OS of the SA-poor (99 patients, 45.2%) and SA-good (120 patients, 54.8%) subgroups were 11.8 and 24.9 months, respectively, (hazard ratio [HR]: 0.21, 95% confidence interval (CI): 0.14–0.31, *p* < 0.0001) for the training dataset, and 14.4 and 19.0 months, respectively, (HR: 0.32, 95% CI: 0.16–0.64, *p* = 0.0001) for the test dataset [25 (40.4%) and 37 (59.6%) patients, Fig. 3A and B]. ESCCPro-SA obtained C-indices of 0.762 and 0.705 for OS evaluation and 0.720 and 0.706 for DFS evaluation for the training and test datasets, respectively. Examples of two patients with ESCC who underwent SA with different ESCCPro-SA scores (0.11 vs 0.95) are presented in Fig. 4A and B. The follow-up data showed that the two patients had distinct OS (of 26.6 and 3.0 months). Details of the evaluation of the secondary endpoint see supplementary A1.

**Fig. 5:**
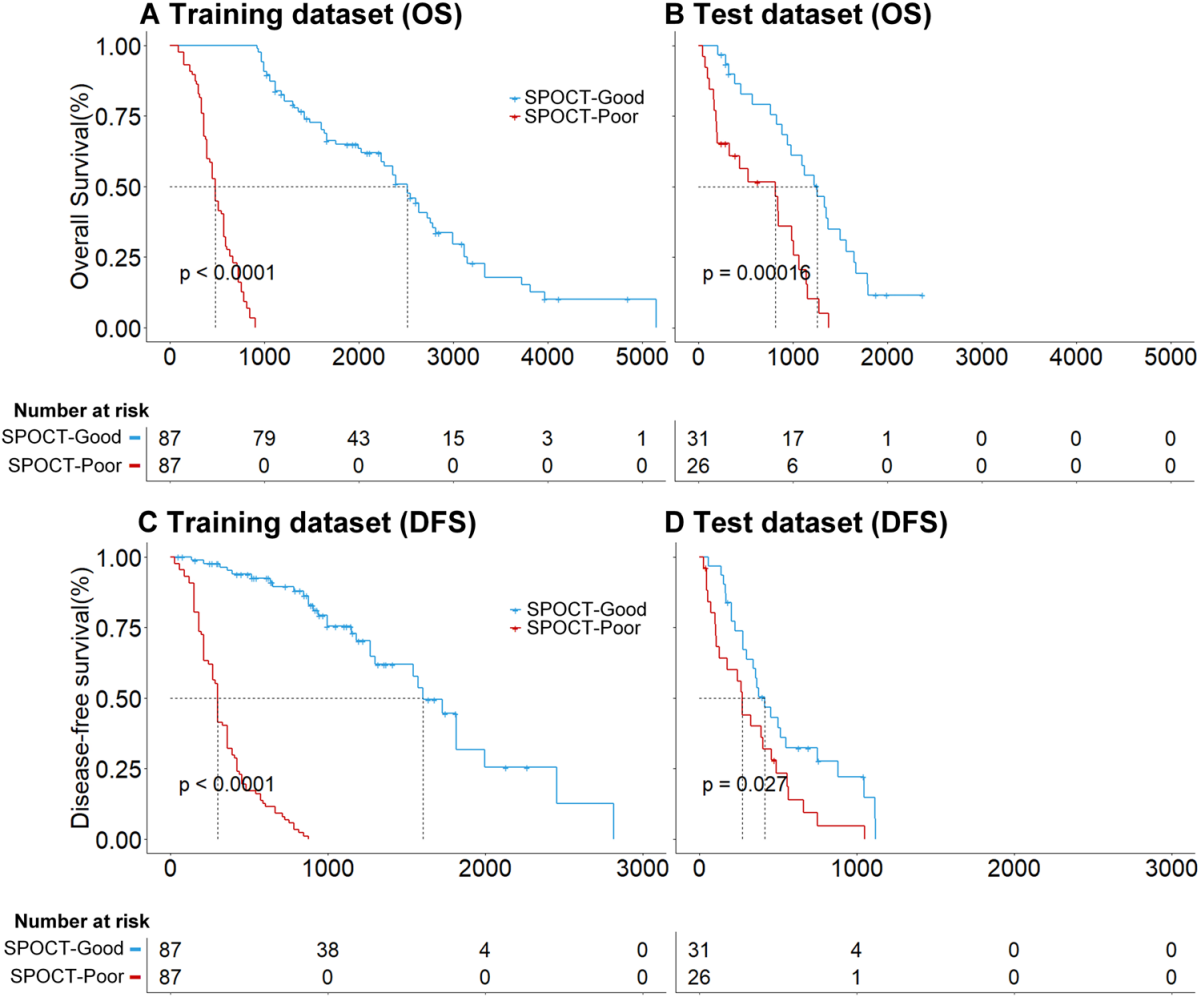
Kaplan–Meier survival curves of the ESCCPro-SPOCT model predicted good and poor outcomes for patients treated with POCT following surgical resection. Significant differences were found between the subgroups for the training and test datasets for both OS (**A**, **B**) and DFS (**C**, **D**) prognosis (*p* < 0.05)

Next, the decision of postoperative adjuvant chemotherapy (POCT) in patients with ESCC was the second task of this study. Following surgical resection, the ESCCPro-SPOCT model was built to evaluate the survival outcomes of patients who underwent SPOCT. The optimal cut-off of 0.2 for the ESCCPro-SPOCT model was determined using X-tile, and patients were classified as SPOCT-poor (high scores) or SPOCT-good (low scores). The median OS of the predicted SPOCT-poor (87 patients, 50.0%) and SPOCT-good (87 patients, 50.0%) subgroups were 16.1 and 66.6 months, respectively, (HR: 0.24, 95% CI: 0.17–0.33, *p* < 0.0001) for the training dataset, and 13.9 and 37.4, respectively, (HR: 0.36, 95% CI: 0.18–0.71, *p* = 0.0001) for the test dataset (26 patients (45.7%) and 31 patients (54.3%), Fig. 5A, B). ESCCPro-SPOCT obtained C-indices of 0.792 and 0.696 for OS evaluation and 0.782 and 0.603 for DFS evaluation for the training and test datasets, respectively. Examples of two patients with ESCC who underwent SPOCT with different ESCCPro-SPOCT scores (0.09 vs 0.75) are presented in Fig. 4C, D. The follow-up data showed that the two patients had distinct OS (of 100.0 and 15.1 months). Detailed results regarding the secondary endpoint see supplementary A1.

When compared with the control group, no significant OS difference was found between the model-predicted SA-poor subgroup and the control cohort in the training (*p* = 0.155) or test (*p* = 0.244, Supplementary Fig. S1) dataset. The predicted SA-good patients had significantly better OS than the control cohort both for the training (*p* < 0.0001) and test datasets (*p* < 0.0001, Supplementary Fig. S1). This finding demonstrates that the ESCCPro can potentially screen patients with ESCC who are unlikely to benefit from surgery. It could potentially support clinical preoperative consideration of alternative therapies and reduce patient burden associated with surgery.

### Clinical assistance evaluation

**Fig. 6:**
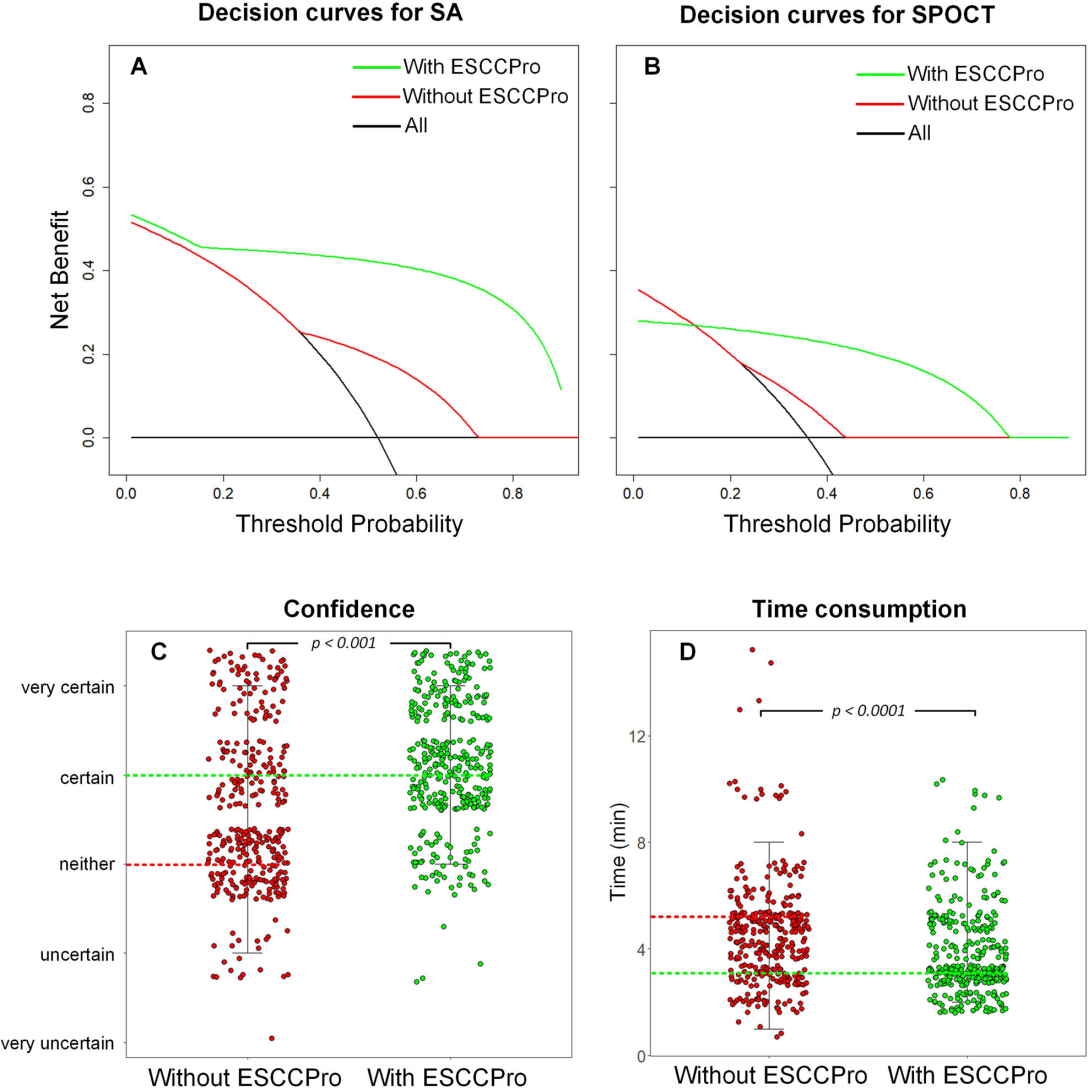
Decision curve analysis of the patients treated with SA (**A**) and SPOCT (**B**). Green lines and red lines denote the results diagnosed by a clinician with 15 years of experience with/without ESCCPro assistance. The black line on the horizontal axis represents “none”. The average diagnostic confidence (with median lines, **C**) and the time consumption per patient (with mean lines, **D**) of all the experts with/without ESCCPro assistance

Decision curve analysis depicted in Fig. 6A revealed that the utilization of the ESCCPro-SA model as an aid in SA decision-making resulted in a significant improvement in clinical management, as indicated by the increased net benefit of patient survival compared to that of the patients where the ESCCPro-SA model was not utilized. In addition, the decision curve analysis depicted in Fig. 6B demonstrated that when there is a probability range of 10–80% for patients with ESCC to achieve good survival through SPOCT (current clinical studies suggest that approximately 50% of the patients with ESCC achieve the desired treatment outcome with SPOCT, thus, falling within this range), incorporating the ESCCPro-SPOCT model as an aid in SPOCT decision-making can enhance the net benefit of patient survival compared to patients where the ESCCPro-SPOCT model was not utilized. Details for further clinical assistance evaluation have been presented in Supplementary A5.

With the model’s assistance, the meantime (with SD) from loading images to logging diagnosis (SA or SPOCT) decreased significantly (*p* < 0.0001) from 4.59 min (± 5.4 min) to 3.62 min (± 4.8 min), and the median confidence improved from “neither” to “certain” (*p* < 0.001, Fig. 6).

## Discussion

A pre-therapy PET/CT image-based deep learning approach, ESCCPro, was developed and externally tested to improve the survival benefit of patients with ESCC treated with SA and SPOCT, as well as to improve the prognostic accuracy, certainty, and efficiency of clinical experts’ decision-making of the two therapeutic approaches. This study comprehensively investigated the ESCCPro as a prognostic prediction tool across SA, SPOCT, and palliative treatment without surgical resection, with endpoint events of OS, DFS, and PFS for patients with ESCC. The results demonstrated that the ESCCPro correlated well with the post-treatment response and long-term prognosis, with lower scores generally indicating better outcomes. In addition, ESCCPro assistance improved the clinician’s accuracy and confidence in practical clinical scenarios, indicating its value in aiding treatment decisions that prolong survival outcomes and achieve better cost-benefit ratios for patients with ESCC.

Although the efficacy of SPOCT has been widely studied [29–32], uncertainty regarding its benefits in patients with ESCC has hampered its administration [10]. Some previous studies have shown that SPOCT with cisplatin and fluorouracil potentially prevents relapse compared to SA [3, 10, 33], whereas other studies found that only half of the treated patients have a complete/partial response [5, 34]. Identifying patients with higher chances of prolonged survival outcomes from additional SPOCT rather than SA could provide substantial evidence for better SPOCT implementation. In this study, half of the patients who may have obtained significantly better survival outcomes from SPOCT were successfully identified by ESCCPro-SPOCT, with significantly higher median OS and DFS in the training dataset. For the test dataset, the predicted SPOCT-good subgroup had significantly better survival outcomes than the SPOCT-poor subgroup. These results potentially inform the screening of suitable patients with ESCC for SPOCT treatment. According to previous studies, SPOCT has demonstrated a median OS of 40.0 months and a 3-year OS rate of 54.0% in patients with ESCC [35]. A median OS of 55.2 months and a 3-year OS rate of 78.0% were seen in the SPOCT-good subgroup identified by ESCCPro-SPOCT in this study. The findings suggest that utilizing the model for determining SPOCT eligibility could have resulted in a substantial improvement in clinical outcomes. Specifically, compared with the median OS of the SPOCT group reported in the previous study (40.0 months) and the median OS of the group that ESCCPro recommended for SPOCT (55.2 months), it could be estimated that when employing ESCCPro for aiding clinical decision-making in future, the survival of patients received SPOCT would be improved.

In clinical practice, patients with ESCC with an appropriate imaging stage for surgical resection are recommended for esophagectomy [36]. This study found that almost half of the SA patients (SA-poor subgroup) clinically suitable for surgical excision did not have a significant improvement in either OS or DFS compared with patients with ESCC in the control cohort. The 2-year OS rate (with median OS) between the predicted SA-good and SA-poor subgroups was 51.0% (24.5 months) vs. 4.8% (12.1 months). Compared with the median OS of 16.6 months and a 2-year OS rate of 39% reported previously [37], the employment of ESCCPro to determine SA eligibility could improve OS by 8 months and the 2-year OS rate by 12.0% for these patients, indicating that ESCCPro-SA could be a valuable tool to support treatment selection. Future clinical applications of ESCCPro may preoperatively identify patients who are unlikely to benefit from surgery. This will provide valuable non-invasive evidence to support timely alternative treatments, reduce surgical burden, and improve survival benefits for patients with ESCC.

There is an insufficient number of clinicians with high ESCC diagnostic expertise, making the creation of accurate clinical treatment plans difficult [38]. When clinicians with varying experience in ESCC treatment were assisted by ESCCPro, prognostic accuracy increased. All seven experts performed well after ESCCPro-SA assistance. Although five experts had significantly improved performance with ESCCPro-SPOCT, the improvements from two mid-level experts were less significant, indicating that implementation of precision SPOCT remains challenging as accurate prognosis is difficult, but ESCCPro-SPOCT could assist. Interestingly, the least and most experienced clinicians benefitted most from ESCCPro support, whereas mid-level experts did not benefit as much. Given that clinicians usually make treatment decisions for ESCC based on PET/CT and baseline characteristics, these results could be used to better distribute ESCC treatment expertise among clinicians.

This study had several limitations. Although the test dataset consisted of patients from two hospitals, incorporating data from additional centers with randomized controlled trials and a fully open-access study protocol would improve the universality and performance of ESCCPro. Different cut-off values were chosen for the two treatments because two models were built for each treatment. Although the strategy of building specific models has been shown to be effective by a previous prognostic study [39], efforts should be made to improve the clinical usability of the ESCCPro models. In addition, further analysis of the impact of other postoperative adjuvant therapies on ESCC (such as radiotherapy and neoadjuvant chemotherapy) survival will provide valuable information for treatment decision-making. Finally, because of the naturally small region of interest of esophageal cancer, the EfficientV2 network (previously proven applicable to lesions of similar sizes) was adopted [12]. Traditional deep learning architectures, such as ResNet and other pretraining models based on higher resolution images [40, 41], may not be able to accurately perceive ESCC tumors. Future specific deep learning prognosis networks and corresponding biological experiments should be designed for ESCC images to further interpret the relationship between deep learning prognostic models and underlying mechanisms such as ESCC molecular pathways.

## Conclusions

The clinical employment of ESCCPro is promising to improve the survival benefit of patients with ESCC treated with SA and SPOCT and to improve the prognostic accuracy, certainty, and efficiency of clinical experts in decision-making regarding choosing the appropriate therapeutic approach. Using pre-therapy non-invasive PET/CT images with end-to-end deep learning approaches could identify high-risk patients, enhance clinical management of controversial treatment strategies, and potentially prolong the survival prognosis of patients with ESCC.

### Supplementary information

ELECTRONIC SUPPLEMENTARY MATERIAL

## Data Availability

The source code of the ESCCPro model is publicly available at the following URL link: https://github.com/OncoAIPro/ESCCPro. The CT, PET, and PET/CT images, the well-trained ESCCPro-SA and ESCCPro-SPOCT models, and the code for model architecture, were made openly accessible: https://data.mendeley.com/datasets/3jbktrwtm4/2.

## Abbreviations

AI: Artificial intelligence
CI: Confidence interval
CT: Computed tomography
DFS: Disease-free survival
ESCC: Esophageal squamous cell carcinomas
ESCCPro: ESCC prognosis model
HR: Hazard ratio
OS: Overall survival
PET: Positron emission tomography
PFS: Progression-free survival
POCT: Postoperative adjuvant chemotherapy
SA: Surgical resection alone
SPOCT: Surgery followed by postoperative adjuvant chemotherapy
