# Supplementary material for: PET/CT deep learning prognosis for treatment decision support in esophageal squamous cell carcinoma
Source: Insights Imaging. 2024 Jun 24;15:161. doi: 10.1186/s13244-024-01737-1 (PMC11196479; doi:10.1186/s13244-024-01737-1)
Supplement: Supplementary file 1 — ELECTRONIC SUPPLEMENTARY MATERIAL [file 13244_2024_1737_MOESM1_ESM.docx]

**PET/CT deep learning prognostication for treatment decision support in esophageal squamous cell carcinoma**

**Supplementary A1**

**
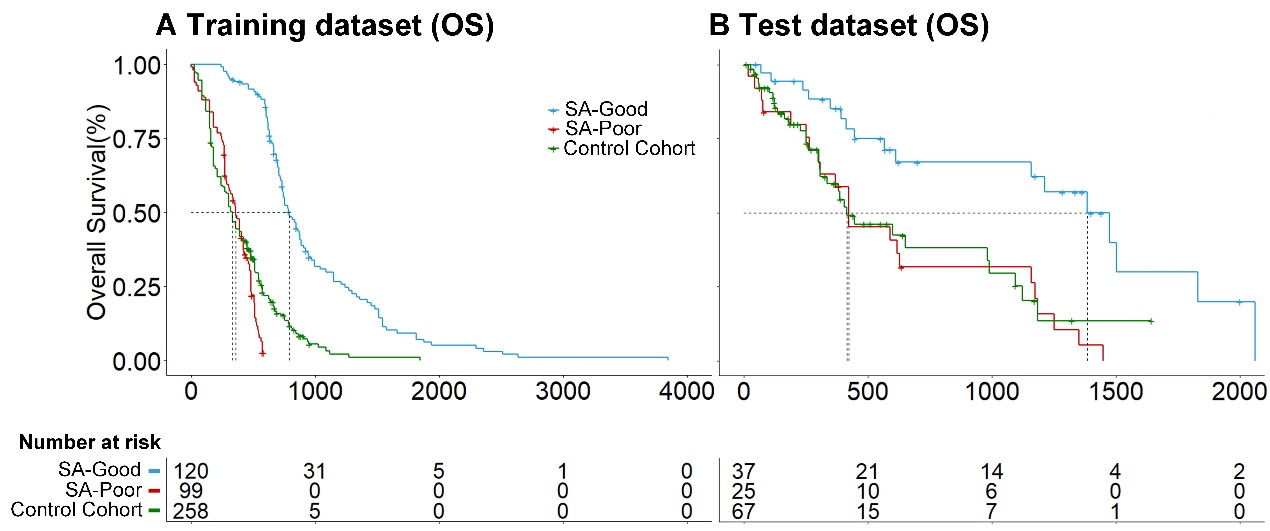
**

**Figure S1.** Comparison of the Kaplan–Meier survival curves of the subgroups of SA-Good and SA-Poor predicted by ESCCPro-SA, and the patients in the control cohort on the training (A) and test (B) datasets. No significant survival difference was found between the control cohort and the SA-Poor subgroup in the training (P=0.155) and test dataset (P=0.244), whereas a significant survival difference was found between the control cohort and the SA-Good subgroup in the training (HR: 0.35, 95%CI: 0.28–0.43, P<0.0001) and test dataset (HR: 0.33, 95% CI: 0.19–0.59, P<0.0001).

Regarding the secondary endpoint for post-treatment response evaluation, when using the same cut-off value as ESCCPro-SA trained on OS, the median DFS of the predicted SA-Poor and SA-Good subgroups were 10.2 and 23.7 months, respectively, for the training dataset (Hazard ratio [HR]: 0.28, 95% confidence interval [CI]: 0.18–0.44, P < 0.0001), and 5.0 and 11.7 months, respectively, (HR: 0.36, 95% CI: 0.18–0.71, P = 0.0001) for the test dataset (Figures 3C and 3D). For SPOCT patients, the median DFS of the ESCCPro-SPOCT-predicted poor and good subgroups were 12.1 and 32.8 months, respectively, for the training dataset (HR: 0.20, 95% CI: 0.13–0.28, P < 0.0001). Those for the test dataset were 9.0 and 12.6 months, respectively (HR: 0.54, 95% CI: 0.30-0.97, P = 0.027, Figures 4C and 4D). The proportional hazard assumption evaluation indicated that the ESCCPro score did not violate the assumption (P = 0.39 and 0.55 on the SA and SPOCT datasets, respectively). In addition, based on the median OS of the predicted SA-Good and SA-Poor patient subgroups and the proportion of patients between the subgroups, the results of the estimated sample sizes by PASS were 151 and 118 for the above two subgroups (power = 0.80). Similarly, the results of the estimated sample sizes by PASS were 88 and 45 for the SPOCT-Good and SPOCT-Poor subgroups (power = 0.80), indicating that the number of patients included in this study was by the statistically estimated sample size requirements.

In addition, for the secondary endpoint, a significant survival difference was found between the model predicted SA-Good patients and the control cohort in both the training and test datasets (P < 0.05, Supplementary material, Figure S2) but not between the model predicted SA-Poor patients and the control cohort (P > 0.05, Supplementary material, Figure S2).


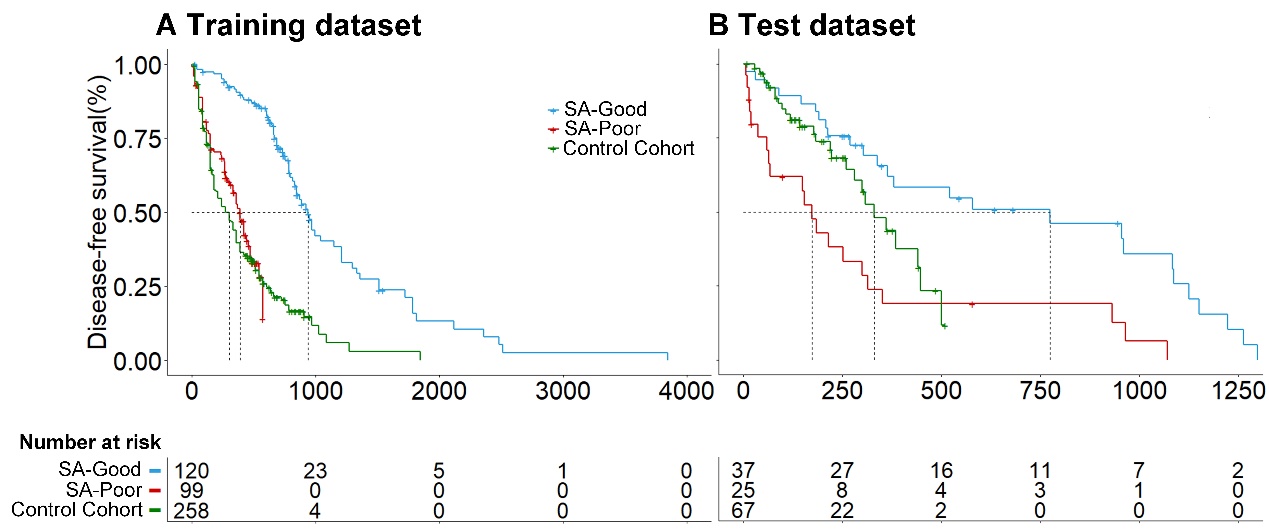


**Figure S2.** Comparison of the Kaplan–Meier survival curves (the secondary endpoint in this study, disease-free survival for SA patients and progression-free survival for control cohort) of the subgroups of SA-Good and SA-Poor predicted by ESCCPro-SA, and the patients in the control cohort in the training (A) and test (B) datasets. No significant survival difference was found between the SA-Poor patients and the patients in the control cohort in the training (P=0.392) and test dataset (P=0.092). However, when compared the survival of SA-Good patients with the control cohort, results indicated significant survival difference was found between the two in the training (HR: 0.31, 95% CI: 0.24–0.41, P < 0.0001) and the test datasets (HR: 0.58, 95% CI: 0.33–1.03, P = 0.0269).


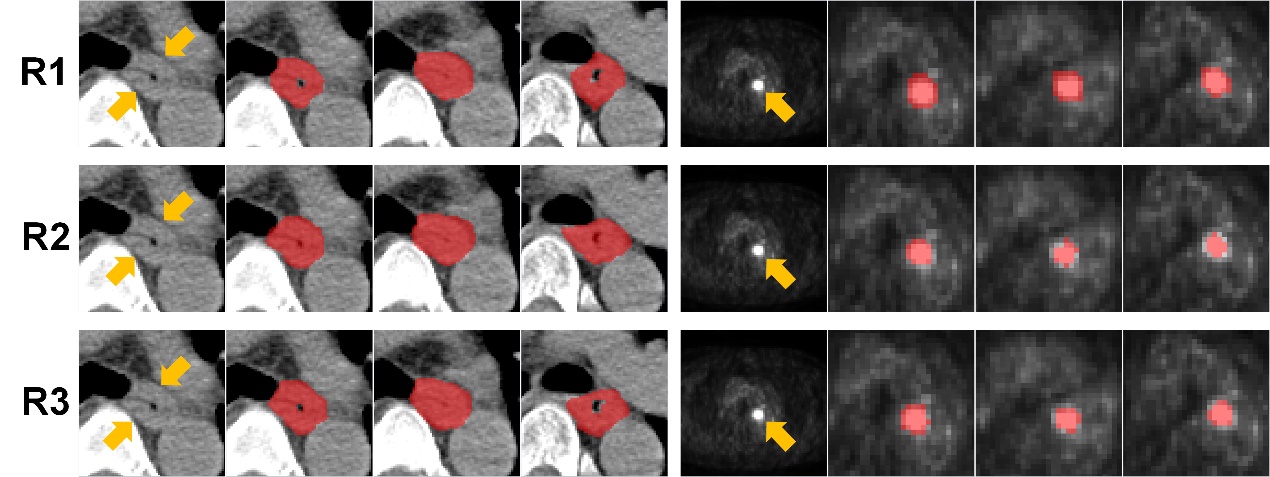


**Figure S3.** Example of manual segmentation of three consecutive slices of the primary lesion of a patient with esophageal squamous cell carcinoma (70-year-old, male, overall survival: 118.2 months) by the three radiologists (R1, R2, and R3) in this study. The first column on the left represents the original CT image, and the first column on the right represents the original PET image of the corresponding slice. Arrows indicate tumor locations.


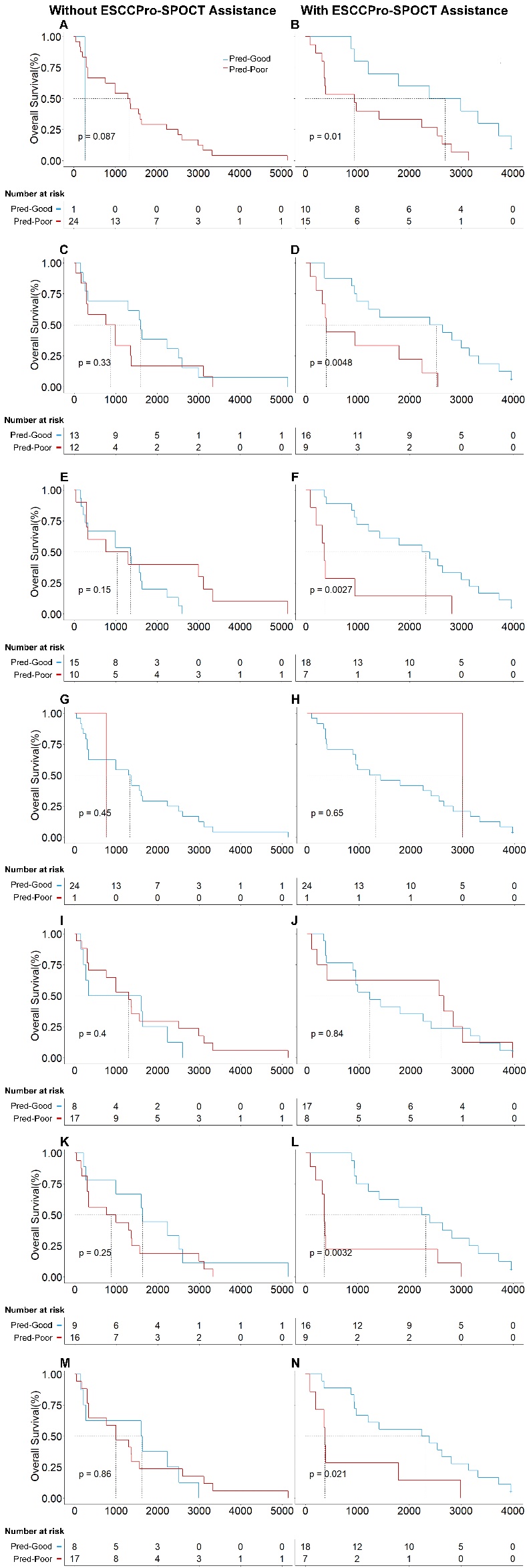


**Figure S4.** Kaplan–Meier curves of the overall survival of the SPOCT patients predicted by the seven experts in the reader study. Figs. A, C, E, G, I, K, and M on the left were the results predicted by the experts without ESCCPro-SPOCT assistance, and Figs. B, D, F, H, J, L, and N on the right were the results predicted by the experts with ESCCPro-SPOCT assistance. Blue line represents the predicted subgroup with good prognosis, and red line represents the predicted subgroup with poor prognosis. P value indicates the statistical significance between the two predicted subgroups.


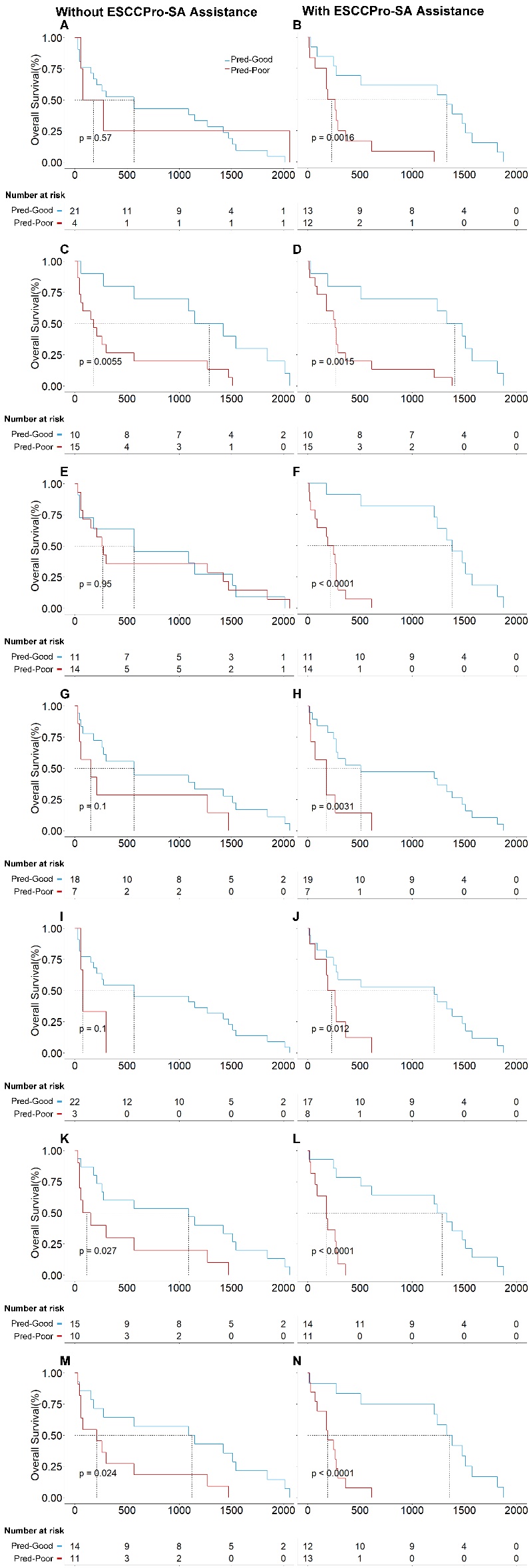


**Figure S5.** Kaplan–Meier curves of the overall survival of SA patients predicted by the seven clinicians in the reader study. Figs. A, C, E, G, I, K, and M on the left represent the results predicted by the experts without ESCCPro-SA assistance, and Figs. B, D, F, H, J, L, and N on the right denote the results predicted by the experts with ESCCPro-SA assistance. Blue line represents the predicted subgroup with good prognosis, and red line represents the predicted subgroup with poor prognosis. P value indicates the statistical significance between the two predicted subgroups.

**Supplementary A2**

**
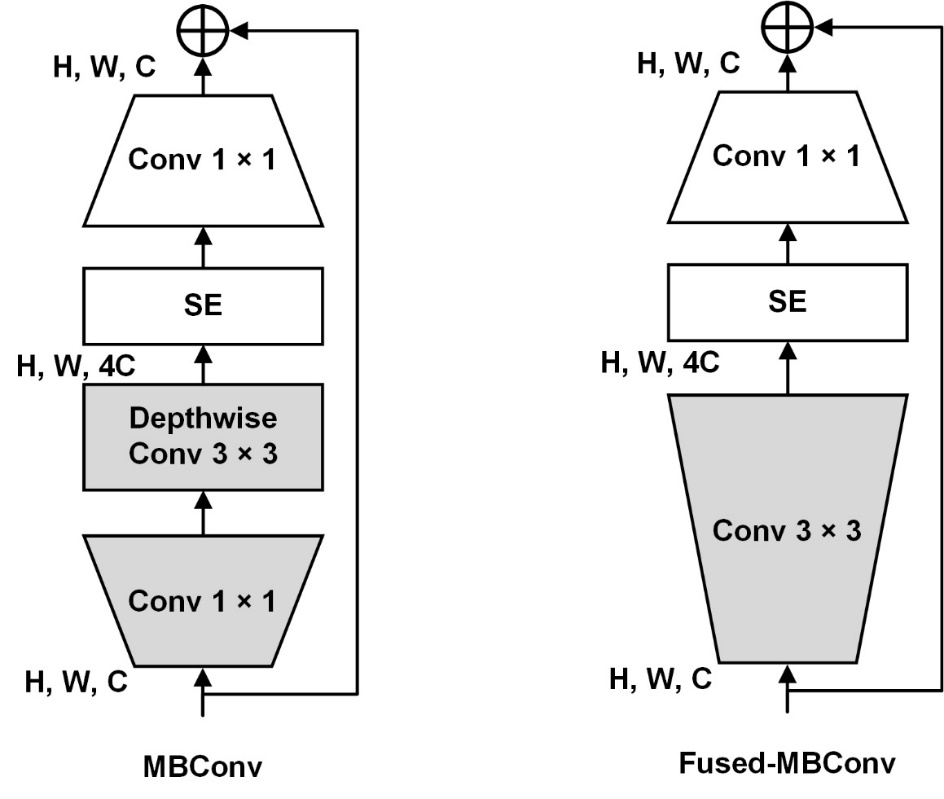
**

**Figure S6.** Structure of the MBConv and Fused-MBConv module in the EfficientNetV2.

The model was implemented using PyTorch (Version: 1.7.1). A computer with one 24 GB TITAN 3090Ti graphic card was used. A batch size of 16 was used, the number of training iterations was set to 250, a weight decay of 1e-5 was added to the model training, the RMSprop optimizer was used with a learning rate calculated by the following Equation (1), the parameters for the RMSprop optimizer were set to α = 0·9, epsilon = 1e-4, momentum = 0·9, and the size of the input CT image was set to 224×224.

$learning rate=batch\_size * cuda\_device\_number * 0.256 / 4096$ (1)

The fully connected layer to output the predicted score of the input image was modified to fit the number of classes in this study. The total training parameters of the ESBP were 22.15 million.

**Table S1.** Pooling functions and hyperparameters of the EfficientNetV2.

|  | **Pooling function** | **Hyperparameters** |
| --- | --- | --- |
| **Model1** | Averaged pooling | Frozen layers = 410, dropout = 0.5, batch size = 16, optimizer= Adam |
| **Model2** | Adaptive attention pooling | Frozen layers = 410, dropout = 0.5, batch size = 16, optimizer= Adam |
| **Model3** | Max pooling | Frozen layers = 410, dropout = 0.5, batch size = 16, optimizer= Adam |
| **Model4** | Averaged pooling | Frozen layers = 300, dropout = 0.3, batch size = 16, optimizer= Adam |
| **Model5** | Adaptive attention pooling | Frozen layers = 300, dropout = 0.3, batch size = 16, optimizer= Adam |
| **Model6** | Max pooling | Frozen layers = 300, dropout = 0.3, batch size = 16, optimizer= Adam |

**Supplementary A3**

All patients included in this study were hospitalized patients collected retrospectively from real-world clinical practice. No patients were selected from previous randomized controlled trials. This study was approved by the ethical review board of each of the included hospitals (Guangdong Provincial People’s Hospital and Zhuhai People's Hospital) and conducted in accordance with the Declaration of Helsinki. The requirement for informed consent was waived owing to the retrospective nature of the study and the analysis of de-identified clinical data.

In this study, the training dataset was used to build the ESCCPro model and the test dataset was used to evaluate the model performance on the data from independent hospitals. The inclusion criteria were patients aged 18–80 years with histologically-confirmed primary ESCC and standardized pretreatment PET/CT scans (within 4 weeks of treatment). Treatments included SA, SPOCT, or no treatment (control cohort, patients with advanced-stage who were unable to undergo surgery and/or chemotherapy). The patients excluded were those who underwent anticancer treatments before the baseline PET/CT scans, had a history of other malignancies, or had incomplete demographic records. For patients received SPOCT, taxane (paclitaxel/docetaxel) and platinum (carboplatin/nedaplatin) combination chemotherapy was administered to 95.2% of patients, whereas the others received paclitaxel and cisplatin combination chemotherapy (4.8%). In most SA cases, patients underwent minimally invasive radical surgery (91.0%), and a minority underwent palliative surgery (9.0%). All surgical procedures were performed following related clinical guidelines [1, 2].

In addition, due to the limited physical tolerance, patients with advanced stage ESCC potentially unable to receive surgery or chemotherapy. Therefore, their survival outcome is generally worse than that of the patients treated with SA and/or SPOCT. The purpose of inclusion of the control group was to compare their survival with that of the patients who received recommended treatment(s) but were unable to benefit from them. In actual clinical scenarios, successfully identifying the ESCC patients who received SA/SPOCT and showed no significant difference in their survival prognosis compared to that of the patients who received no treatment holds the potential to suggest that these patients should avoid undergoing surgical resection, thereby reducing the treatment costs and clinical risks for the patients.

The median follow-up period (with [inter-quartile range]) was 35.5 [11.5, 68.3] months and did not differ significantly between the training and test datasets (P = 0.125) for the SA (31.5, [12.6, 66.1] months), SPOCT (33.1, [15.0, 70.2] months), and control group (26.8, [10.1, 62.7] months) patients.

Sample size evaluation was performed using PASS (Version 21.0.3, NCSS, LLC, UTAH). Dice coefficient was used to evaluate inter-observer and intra-observer biases in ESCC manual segmentation, and an average Dice coefficient > 95% was considered robust [3]. Harrell's C-index (95% confidence interval) was used to measure the prognostic performance of ESCCPro. Kaplan–Meier survival curves with log-rank tests were used to analyze difference in survival between subgroups, and a hazard ratio with a 95% CI was used to evaluate differences in the ESCCPro subgroup survival. The assumption of Cox proportional hazards was also evaluated. X-tile was used to determine the optimal cut-off of the final score [4], and when the cut-off value was determined on the training dataset, it was locked and applied to the test dataset. In the clinical assistance evaluation experiment, the agreement between diagnoses made by each expert with and without the assistance of ESCCPro was calculated using Fleiss’ κ [5]. Decision curve analysis was used to evaluate the difference between the predictions by comparing the diagnostic accuracy in the first (without ESCCPro assistance) and last half (with ESCCPro assistance) of the group of patients [6, 7].

**Supplementary A4**

PET/CT examinations were performed from the calvarium to mid-femur (training dataset: Discovery MI, GE Healthcare; test dataset: Sensation Biograph Somatom 16HR PET/CT, SIEMENS; and Gemni TF 64 PET/CT, Philips). Patients were instructed to fast for at least 6h prior to their PET/CT scans, ensuring a normal blood glucose level. CT scans were firstly acquired for anatomical localization and attenuation correction, and PET data were subsequently acquired in 3D mode. The acquisition parameters for CT scan were: 120 kV, 60–80 mAs, field of view 600 mm. CT scan images were reconstructed using a filtered back projection with 1.25–5 mm thickness and 512×512 matrix. The acquisition time of PET scanning was 1 min per bed position, and the scanning range was covered with 5- to 7-bed positions. Then, the scan results were reconstructed using the super iterative algorithm. Two radiologists with more than 10 years of experience performed manual segmentation of all primary ESCC tumors on the PET and CT images slice-by-slice. For PET images, the radiologists manually identified the boundary of the metabolically active tumor and delineated it using ITK-SNAP software (version 3.6.0). For CT images, ESCC lesions were retrieved from the calvarium to the mid-femur. Tumor boundaries were determined by comparing the locations with PET images, and ITK-SNAP was used for manual delineation.

In this study, a log-rank test is used for survival analysis, and the ESCCPro-SA model is proposed to categorize the SA patients into two subgroups. Based on all the included SA patients, the median OS of the SA-good patients predicted by ESCCPro-SA is 22.3 months, and that of the other subgroup is 12.1 months (without censored data). The ratio of the number of patients in the two subgroups is 0.77 in this study. Based on the above conditions, the results of the estimated sample sizes by PASS are 151 and 118 for the two subgroups (power = 0.80), with a two-sided alpha of 0.05. Regarding the SPOCT patients, the median OS of the SPOCT-good patients predicted by ESCCPro-SPOCT is 45.5 months, and that of the other subgroup is 15.5 months (without censored data). The ratio of the number of patients in the two subgroups is 0.95 in this study. Based on the above conditions, the results of the estimated sample sizes by PASS are 88 and 45 for the two subgroups (power = 0.80), with a two-sided alpha of 0.05.

**Supplementary A5**

Dice coefficients of 97.1% and 98.3% demonstrated excellent inter-observer and intra-observer agreement, respectively, for tumor segmentation (Supplementary material, Figure S3). Analysis of variance showed that there was no statistically significant difference in ESCCPro scores calculated based on two rounds of segmented images (P = 0.70). The decision assistance study results showed that the mean C-index of all clinicians was 0.579 (range, 0.512–0.662) without assistance from ESCCPro-SA, which improved to 0.671 when assisted by the ESCCPro-SA model (range, 0.630–0.731). In addition, the decision curve analysis depicted in Figure 6A revealed that the utilization of the ESCCPro-SA model as an aid in SA decision-making resulted in a significant improvement in clinical management, as indicated by the increased net benefit of patient survival compared to that of the patients where the ESCCPro-SA model was not utilized. For patients treated with SPOCT, the results indicated an average C-index of 0.535 (range, 0.510–0.589) without the model, which improved to 0.602 (range, 0.525–0.673) with ESCCPro-SPOCT assistance. The model improved the accuracy of the clinical decision-making of clinicians with approximately 2 years of experience (C-index from 0.550 to 0.651 on average), and those with >10 years of experience (C-index from 0.585 to 0.675 on average); clinicians with mid-level experience improved the least (C-index from 0.541 to 0.576 on average). The diagnostic agreement between the seven experts improved from 0.259 to 0.511 with model assistance. In addition, the decision curve analysis depicted in Figure 6B demonstrated that when there is a probability range of 10–80% for patients with ESCC to achieve good survival through SPOCT (current clinical studies suggest that approximately 50% of the patients with ESCC achieve the desired treatment outcome with SPOCT [5-9], thus, falling within this range), incorporating the ESCCPro-SPOCT model as an aid in SPOCT decision-making can enhance the net benefit of patient survival compared to patients where the ESCCPro-SPOCT model was not utilized. This finding suggests that utilizing the ESCCPro-SPOCT model as a support tool for SPOCT decision-making has the potential to improve the clinical management of patients with ESCC.

For diagnosis of surgery implementation, four of the seven experts’ diagnoses indicated no significant difference, which was reduced to none with ESCCPro-SA assistance (Supplementary material, Figure S5). In addition, there was no significant difference in OS between the subgroups from all seven experts’ diagnoses on whether SPOCT should be performed; this was reduced to two of seven experts with ESCCPro-SPOCT assistance (Supplementary material, Figure S4). The above findings indicate the ESCCPro models help improve the accuracy of the clinician's decision making on whether to perform SA and SPOCT on the patients with ESCC.

**Supplementary A6**

***RNA correlation analysis***

Based on the available high-throughput RNA sequencing data for the enrolled patients with ESCC, an RNA cohort was established for correlation analysis of the scores of the ESCCPro model. Details of the RNA sequencing is presented below. The top 75% of all RNA genes was first retained based on the median absolute deviation score (> 0.01). A list of RNA genes significantly associated with the overall survival of patients, and another list of RNA genes significantly associated with the ESCCPro scores of patients were then identified using Pearson correlation analysis. The consistency of the two RNA gene lists was used to further corroborate the credibility of the ESCCPro scores for prognostic assessment of patients with ESCC.

***RNA sequencing***

For RNA sequencing, 62 paraffin-embedded ESCC specimens at Guangdong Provincial People’s Hospital from 2015 to 2021 were enrolled. Total RNA extraction was performed as previously described [8]. After assessing RNA quality and integrity, it was fragmented according to the DV200 value. Then, eligible RNA fragments were reverse-transcribed and synthesized into complementary DNA, followed by preparing a strand-specific library by the NEBNext® Ultra™ II Directional RNA Library Prep Kit for Illumina®. An AmoyDx® FFPE RNA Extraction Kit was used for total RNA extraction. DV200 value was estimated with an Agilent 2100 Bioanalyzer System. Library size was assessed using an Agilent 2100 Bioanalyzer, and libraries were sequenced on an Illumina NovaSeq 6000 instrument (Illumina). Captured libraries were sequenced through 2×150 bp paired end reads following sophisticated data quality control parameters. Detailed analysis was shown in the previous study [9].

***Results of RNA correlation analysis***

The RNA sequencing data of 62 enrolled patients was available for this study. A total of 33,953 RNA genes per patient were included. When selecting the top 75% of genes based on the median absolute deviation score (> 0.01), 19,667 genes were retained. Based on the Pearson correlation analysis, 173 genes were significantly correlated with OS (|Pearson correlation coefficient| > 0.3 and P < 0.05), and 154 genes were significantly correlated with the ESCCPro score. The total number of overlapping genes in the two gene lists was 53, which accounted for 30.64% and 34.41% of the OS-correlated and ESCCPro-correlated gene lists, respectively. This result suggests that more than 30% of the RNA genes associated with the ESCCPro score are those which are significantly associated with survival prognosis of patients with ESCC. Detailed results of the Pearson correlation analysis are presented in Tables S2 and S3 below.

**Table S2.** The RNA genes showed significantly associated with (|Pearson correlation coefficient| > 0.3 and P < 0.05) overall survival among the 62 patients with RNA sequencing data in this study.

| Gene name | Pearson correlation coefficient | P value |
| --- | --- | --- |
| ENPP7P12 | 0.520191807 | 1.47E-05 |
| HBG2 | 0.500307812 | 3.46E-05 |
| IRF4 | 0.466699117 | 0.000132 |
| ZFPL1 | 0.466335212 | 0.000133 |
| TRIP4 | 0.457431327 | 0.000186 |
| MTMR14 | 0.450167709 | 0.000241 |
| INPP5D | 0.449094575 | 0.000251 |
| SNUPN | 0.446323664 | 0.000277 |
| XDH | 0.44539245 | 0.000286 |
| SLC25A39 | 0.433186058 | 0.000437 |
| ADAM8 | 0.430561221 | 0.000477 |
| CYBC1 | 0.428017937 | 0.00052 |
| KLK6 | 0.427726099 | 0.000525 |
| KANSL1 | 0.42526566 | 0.00057 |
| WDR4 | 0.422843704 | 0.000617 |
| POTEE | 0.417253693 | 0.000741 |
| RN7SKP70 | 0.416584697 | 0.000757 |
| S100A7 | 0.414965759 | 0.000798 |
| PAPSS2 | 0.414510763 | 0.00081 |
| LOC101928622 | 0.412248368 | 0.00087 |
| CHKB-DT | 0.412195042 | 0.000872 |
| SGK1 | 0.410689614 | 0.000915 |
| PDXDC2P | 0.410418767 | 0.000923 |
| WDR87BP | 0.409272644 | 0.000957 |
| LOC100419700 | 0.390126805 | 0.001721 |
| LINC02166 | 0.390126805 | 0.001721 |
| LOC107985131 | 0.390126805 | 0.001721 |
| SMAD5 | -0.389711968 | 0.001742 |
| PLXND1 | 0.387204701 | 0.001877 |
| ADAMTSL4-AS1 | 0.386964466 | 0.00189 |
| RNF103-CHMP3 | 0.386512894 | 0.001916 |
| RNF125 | 0.386137436 | 0.001937 |
| PDXDC2P-NPIPB14P | 0.385052873 | 0.001999 |
| LINC01679 | 0.385024118 | 0.002001 |
| CCL11 | 0.384511445 | 0.002031 |
| MT4 | 0.383442659 | 0.002096 |
| PPP2R3A | 0.38340071 | 0.002098 |
| COX17 | 0.382585428 | 0.002149 |
| SFTPB | 0.382064504 | 0.002182 |
| POU2AF1 | 0.381387699 | 0.002225 |
| HCLS1 | 0.380004543 | 0.002316 |
| NT5C1B-RDH14 | 0.37992092 | 0.002321 |
| RPS6KB1 | 0.37953721 | 0.002347 |
| NFAM1 | 0.379230995 | 0.002368 |
| NCSTN | 0.374047227 | 0.002746 |
| CTNNBL1 | 0.374017297 | 0.002748 |
| STAB1 | 0.372761132 | 0.002848 |
| POLM | 0.372422959 | 0.002875 |
| CEBPA-DT | 0.371856247 | 0.002921 |
| RAB31 | 0.37047888 | 0.003036 |
| TMEM129 | -0.369887877 | 0.003087 |
| NRF1 | 0.369416814 | 0.003128 |
| MIR4435-2HG | 0.369159622 | 0.00315 |
| DDX5 | 0.368401714 | 0.003218 |
| NOTCH2NLB | 0.367675826 | 0.003283 |
| CENPP | 0.36743149 | 0.003306 |
| FAM156A | 0.367269863 | 0.00332 |
| S100A9 | 0.365677623 | 0.00347 |
| PDZK1IP1 | 0.365100502 | 0.003526 |
| FGR | 0.364635398 | 0.003571 |
| NRBP1 | 0.364360786 | 0.003598 |
| HBA1 | 0.364341254 | 0.0036 |
| MAGEA4 | 0.362128659 | 0.003825 |
| PARVG | 0.36207273 | 0.003831 |
| UBTF | 0.361916405 | 0.003847 |
| TTC7A | 0.3611304 | 0.003931 |
| ACAD9 | 0.361126894 | 0.003931 |
| TP73-AS1 | 0.360888337 | 0.003957 |
| RNF149 | 0.36012283 | 0.00404 |
| SAT1 | 0.360016403 | 0.004051 |
| COTL1 | 0.35894124 | 0.004171 |
| LOC105378429 | 0.358004253 | 0.004278 |
| LOC440910 | 0.356420487 | 0.004464 |
| XPNPEP1 | 0.349644327 | 0.005343 |
| MLKL | 0.349307357 | 0.005391 |
| G6PC3 | -0.34857244 | 0.005495 |
| PLIN3 | 0.348390695 | 0.005522 |
| HIF1A-AS3 | 0.348219534 | 0.005546 |
| N4BP2L2-IT2 | 0.344885106 | 0.006049 |
| IL4R | 0.344592001 | 0.006095 |
| ZNF8 | -0.344570405 | 0.006098 |
| BPIFB2 | 0.344547681 | 0.006102 |
| UBE2G2 | 0.344343469 | 0.006134 |
| ISY1 | 0.344309632 | 0.006139 |
| TES | 0.344259602 | 0.006147 |
| ZNF276 | 0.344021836 | 0.006185 |
| XRRA1 | 0.343034313 | 0.006344 |
| LPXN | 0.342663233 | 0.006405 |
| VSIR | 0.342429059 | 0.006444 |
| TMEM255A | -0.342240045 | 0.006475 |
| SRGN | 0.341982923 | 0.006518 |
| MEI1 | 0.341528827 | 0.006594 |
| CRTC2 | 0.341353977 | 0.006624 |
| CHI3L1 | 0.341269896 | 0.006638 |
| SNRNP25 | 0.340913627 | 0.006698 |
| INPPL1 | 0.340675535 | 0.006739 |
| SLC9A3P2 | 0.340532685 | 0.006764 |
| TRIM11 | 0.340115687 | 0.006836 |
| PSTPIP2 | 0.340098907 | 0.006839 |
| DZANK1 | -0.339022573 | 0.007029 |
| H3C6 | 0.335860692 | 0.007613 |
| GMIP | 0.335217234 | 0.007737 |
| ARHGAP18 | 0.334980468 | 0.007783 |
| SEPTIN7P3 | 0.334891964 | 0.007801 |
| CYHR1 | -0.334619168 | 0.007854 |
| TXNIP | 0.334344068 | 0.007908 |
| PEX11B | 0.332226325 | 0.008337 |
| HOXB-AS3 | 0.331758641 | 0.008434 |
| HBB | 0.33160933 | 0.008466 |
| FRMD8 | 0.331505882 | 0.008487 |
| VASP | 0.330679883 | 0.008663 |
| STUB1 | 0.330017231 | 0.008806 |
| CSF3R | 0.32489422 | 0.009982 |
| EXOSC7 | 0.324843691 | 0.009994 |
| TPM4 | 0.324833347 | 0.009996 |
| PBLD | -0.324735048 | 0.01002 |
| DOCK2 | 0.324512089 | 0.010075 |
| CD79A | 0.323763311 | 0.010259 |
| XKR8 | 0.322195809 | 0.010654 |
| SLCO2B1 | 0.321843247 | 0.010745 |
| LCN1P1 | 0.321759453 | 0.010767 |
| DOP1B | 0.321625162 | 0.010801 |
| PLK2 | 0.321481753 | 0.010839 |
| LOC100422274 | 0.321211036 | 0.010909 |
| EIF2B1 | 0.321159938 | 0.010923 |
| CDV3 | 0.321123741 | 0.010932 |
| ITSN2 | 0.320999831 | 0.010965 |
| KDM5D | 0.320383559 | 0.011128 |
| CORO1A | 0.32005572 | 0.011215 |
| ISG20 | 0.319777176 | 0.01129 |
| EFHD2 | 0.319725856 | 0.011304 |
| ZNF529-AS1 | 0.319515709 | 0.011361 |
| DYRK4 | -0.319217668 | 0.011442 |
| AFAP1L1 | 0.319020058 | 0.011496 |
| TTC27 | 0.318766837 | 0.011565 |
| TMEM67 | -0.318456456 | 0.011651 |
| CD163 | 0.318401734 | 0.011666 |
| SPATA6L | -0.318273798 | 0.011702 |
| TCHP | 0.317934428 | 0.011796 |
| RBBP5 | 0.317867231 | 0.011815 |
| CLEC17A | 0.317397598 | 0.011947 |
| TNFAIP3 | 0.31739716 | 0.011947 |
| KHNYN | 0.317262164 | 0.011986 |
| RREB1 | 0.316403132 | 0.012232 |
| DNASE1L3 | 0.316258907 | 0.012273 |
| CALM3 | 0.316193289 | 0.012292 |
| ODR4 | 0.315942314 | 0.012365 |
| CYB5R4 | 0.315578479 | 0.012472 |
| MYO1G | 0.31542384 | 0.012517 |
| C8orf33 | -0.315333828 | 0.012544 |
| UBXN2A | 0.315256395 | 0.012567 |
| LOC100131635 | 0.315009897 | 0.01264 |
| RAB7A | 0.314574407 | 0.01277 |
| PTPRB | 0.311310147 | 0.013781 |
| RASA2 | 0.3111123 | 0.013845 |
| XG | -0.310926657 | 0.013905 |
| GAGE4 | 0.310839141 | 0.013933 |
| CEACAM22P | 0.310797694 | 0.013946 |
| SLC39A4 | -0.310661136 | 0.013991 |
| CREB3L2 | 0.310366159 | 0.014087 |
| ZYX | 0.31014799 | 0.014158 |
| KAT5 | 0.306943323 | 0.015242 |
| ZNF841 | 0.306781091 | 0.015299 |
| ZNF638 | 0.306320874 | 0.015461 |
| NFKBIA | 0.306274113 | 0.015477 |
| ITPRIPL2 | 0.306196379 | 0.015505 |
| ALG1 | 0.306027955 | 0.015564 |
| RTL6 | -0.305945728 | 0.015594 |
| MTO1 | 0.305921929 | 0.015602 |
| KCTD17 | -0.305652822 | 0.015698 |
| WASH9P | 0.305546874 | 0.015736 |
| ENTR1 | -0.304773383 | 0.016016 |
| FKBP5 | 0.301890476 | 0.017096 |

**Table S3.** The RNA genes showed significantly associated with (|Pearson correlation coefficient| > 0.3 and P < 0.05) the output score of ESCCPro among the 62 patients with RNA sequencing data in this study.

| Gene name | Pearson correlation coefficient | P value |
| --- | --- | --- |
| SLC25A39 | -0.46731 | 0.000129 |
| MAGEA4 | -0.42532 | 0.000569 |
| SMAD5 | 0.416864 | 0.000751 |
| SFTPB | -0.41394 | 0.000825 |
| FAM156A | -0.41023 | 0.000928 |
| KLF6 | -0.38972 | 0.001742 |
| ZBTB33 | 0.388024 | 0.001832 |
| PIN4 | 0.383962 | 0.002064 |
| CDH5 | -0.3835 | 0.002092 |
| CSF3R | -0.38184 | 0.002196 |
| SLC18B1 | -0.37794 | 0.002457 |
| FKBP5 | -0.37736 | 0.002499 |
| SUFU | -0.37397 | 0.002752 |
| KAT5 | -0.37238 | 0.002878 |
| PDCD11 | -0.3702 | 0.00306 |
| TMEM255A | 0.370028 | 0.003075 |
| STUB1 | -0.37002 | 0.003076 |
| SOD2 | -0.36956 | 0.003115 |
| MGME1 | 0.369493 | 0.003121 |
| SPIRE1 | -0.3694 | 0.003129 |
| RREB1 | -0.36922 | 0.003145 |
| POLM | -0.3688 | 0.003182 |
| PPIL2 | 0.365838 | 0.003455 |
| DUSP22 | -0.36427 | 0.003607 |
| ETS2 | -0.36326 | 0.003708 |
| NCSTN | -0.36281 | 0.003754 |
| NUCB1 | -0.36113 | 0.003931 |
| TCN1 | -0.36081 | 0.003965 |
| CDC7 | 0.360217 | 0.004029 |
| XPNPEP1 | -0.35969 | 0.004087 |
| CYHR1 | 0.359072 | 0.004156 |
| KREMEN1 | 0.359046 | 0.004159 |
| RBBP9 | 0.358095 | 0.004267 |
| INCENP | -0.35808 | 0.004269 |
| GABPB2 | 0.357771 | 0.004305 |
| NF1 | 0.357 | 0.004395 |
| ADAM8 | -0.35676 | 0.004423 |
| TOPORS | 0.356735 | 0.004426 |
| CXCL1 | -0.35499 | 0.004638 |
| S100A7 | -0.35496 | 0.004641 |
| PLIN3 | -0.35494 | 0.004645 |
| SPATA6L | 0.35035 | 0.005245 |
| CD36 | -0.34593 | 0.005887 |
| IL4R | -0.34576 | 0.005913 |
| HIF1A-AS3 | -0.34203 | 0.00651 |
| PLXNA1 | -0.34192 | 0.006528 |
| HIP1R | -0.34161 | 0.00658 |
| RPS6KB1 | -0.34072 | 0.006731 |
| MRPL53 | -0.34024 | 0.006815 |
| DHX38 | -0.34016 | 0.006828 |
| CRNKL1 | 0.339928 | 0.006869 |
| MRPL57 | 0.339799 | 0.006892 |
| HNF4G | 0.339797 | 0.006892 |
| TNFAIP3 | -0.33957 | 0.006931 |
| TNKS1BP1 | -0.339 | 0.007034 |
| CASP6 | 0.338972 | 0.007038 |
| UGT1A1 | 0.338971 | 0.007038 |
| KLK6 | -0.338 | 0.007214 |
| LOC101928725 | -0.33466 | 0.007846 |
| LINC01537 | -0.33446 | 0.007885 |
| RAB7A | -0.33437 | 0.007903 |
| VPS37C | -0.33399 | 0.007979 |
| UBE2G2 | -0.33393 | 0.007992 |
| KDM4B | -0.32911 | 0.009005 |
| XDH | -0.3291 | 0.009007 |
| TCIRG1 | -0.32908 | 0.009012 |
| KIZ | 0.32883 | 0.009067 |
| USP19 | -0.32821 | 0.009205 |
| DNAJA2 | -0.32801 | 0.009251 |
| TRIM17 | -0.32779 | 0.009302 |
| RNF103-CHMP3 | -0.3277 | 0.009323 |
| NFAM1 | -0.32752 | 0.009363 |
| TRIP4 | -0.32741 | 0.009387 |
| ZFPL1 | -0.32713 | 0.009454 |
| FAH | -0.32697 | 0.00949 |
| SRSF2 | -0.32689 | 0.009508 |
| NUP88 | 0.326403 | 0.009622 |
| C8orf33 | 0.325958 | 0.009727 |
| NIPSNAP3A | 0.325817 | 0.00976 |
| FAF2 | -0.32578 | 0.009768 |
| DBT | 0.325532 | 0.009828 |
| TMEM129 | 0.325279 | 0.009889 |
| PNPLA8 | -0.32506 | 0.009942 |
| GRPEL2 | -0.32473 | 0.01002 |
| ESAM | -0.32441 | 0.010101 |
| SLC39A8 | -0.32417 | 0.010158 |
| RCAN3 | 0.323863 | 0.010234 |
| TRMO | 0.323737 | 0.010265 |
| HBG2 | -0.32318 | 0.010403 |
| RAP2C-AS1 | -0.32301 | 0.010448 |
| TRMT10A | 0.322975 | 0.010456 |
| PCF11 | 0.32292 | 0.01047 |
| PTPRB | -0.32237 | 0.010608 |
| SLA | -0.31822 | 0.011717 |
| NFKBIA | -0.31802 | 0.011772 |
| FAM87A | -0.3176 | 0.01189 |
| GLT8D1 | 0.317566 | 0.0119 |
| S100A9 | -0.31724 | 0.011991 |
| UGT1A6 | 0.317002 | 0.01206 |
| SPECC1 | -0.31696 | 0.012072 |
| FGR | -0.31688 | 0.012095 |
| RGCC | -0.31295 | 0.013265 |
| ENTR1 | 0.3125 | 0.013405 |
| TNIP1 | -0.31236 | 0.013448 |
| PPARD | -0.31227 | 0.013478 |
| RBAK | -0.31214 | 0.013518 |
| UBE3B | -0.31204 | 0.013549 |
| LOC100286922 | 0.312032 | 0.013552 |
| ARFGAP1 | -0.31198 | 0.013567 |
| ZNF8 | 0.311959 | 0.013575 |
| TSKU | 0.311944 | 0.01358 |
| CD163 | -0.31178 | 0.013632 |
| EFHD2 | -0.30763 | 0.015003 |
| RBIS | 0.307206 | 0.015151 |
| C2CD5 | 0.306704 | 0.015326 |
| SNX33 | -0.30637 | 0.015443 |
| ZNF740 | 0.306183 | 0.015509 |
| ZYX | -0.30616 | 0.015516 |
| NSRP1 | 0.306093 | 0.015541 |
| SYT1 | 0.306072 | 0.015549 |
| CDKL2 | -0.30607 | 0.015549 |
| RBM12B | 0.306051 | 0.015556 |
| LOC440910 | -0.30583 | 0.015633 |
| ATP6V0D1 | -0.30583 | 0.015633 |
| RNF149 | -0.30581 | 0.015641 |
| MPP1 | -0.30543 | 0.01578 |
| FRMD8 | -0.30523 | 0.015849 |
| HAGLR | -0.30514 | 0.015883 |
| TREX1 | 0.305134 | 0.015885 |
| CNTNAP4 | -0.30503 | 0.015922 |
| TP73 | 0.304996 | 0.015935 |
| XKR8 | -0.30499 | 0.015936 |
| BRD3 | 0.304958 | 0.015949 |
| DHX40P1 | 0.304829 | 0.015996 |
| DZANK1 | 0.304721 | 0.016035 |
| ARHGEF19 | 0.304713 | 0.016038 |
| CASK | 0.304207 | 0.016223 |
| PBDC1 | 0.304203 | 0.016225 |
| IRAK3 | -0.30408 | 0.016271 |
| TRIM11 | -0.30387 | 0.016348 |
| WTAP | -0.30381 | 0.016368 |
| FAM122A | 0.303728 | 0.0164 |
| ITGA5 | -0.3037 | 0.016412 |
| TTC37 | -0.30348 | 0.016494 |
| TAF9B | 0.30347 | 0.016497 |
| SLC25A42 | 0.303339 | 0.016546 |
| TCHP | -0.30319 | 0.016602 |
| CDC40 | 0.30263 | 0.016813 |
| CHCHD4 | 0.302556 | 0.016841 |
| HMOX1 | -0.3023 | 0.016938 |
| H3C6 | -0.3022 | 0.016976 |
| ITSN2 | -0.30183 | 0.017119 |
| LRRCC1 | 0.301362 | 0.0173 |
| PDXDC2P | -0.30095 | 0.017461 |

**References**

[1] J.A. Ajani, T.A. D'Amico, D.J. Bentrem, D. Cooke, C. Corvera, P. Das, P.C. Enzinger, T. Enzler, F. Farjah, H. Gerdes, M. Gibson, P. Grierson, W.L. Hofstetter, D.H. Ilson, S. Jalal, R.N. Keswani, S. Kim, L.R. Kleinberg, S. Klempner, J. Lacy, F. Licciardi, Q.P. Ly, K.A. Matkowskyj, M. McNamara, A. Miller, S. Mukherjee, M.F. Mulcahy, D. Outlaw, K.A. Perry, J. Pimiento, G.A. Poultsides, S. Reznik, R.E. Roses, V.E. Strong, S. Su, H.L. Wang, G. Wiesner, C.G. Willett, D. Yakoub, H. Yoon, N.R. McMillian, L.A. Pluchino, Esophageal and Esophagogastric Junction Cancers, Version 2.2023, NCCN Clinical Practice Guidelines in Oncology, Journal of the National Comprehensive Cancer Network : JNCCN, 21 (2023) 393-422.

[2] F. Lordick, C. Mariette, K. Haustermans, R. Obermannová, D. Arnold, Oesophageal cancer: ESMO Clinical Practice Guidelines for diagnosis, treatment and follow-up, Annals of oncology : official journal of the European Society for Medical Oncology, 27 (2016) v50-v57.

[3] J. Zhu, X. Chen, B. Yang, N. Bi, T. Zhang, K. Men, J. Dai, Evaluation of Automatic Segmentation Model With Dosimetric Metrics for Radiotherapy of Esophageal Cancer, Frontiers in oncology, 10 (2020) 564737.

[4] R.L. Camp, M. Dolled-Filhart, D.L. Rimm, X-tile: a new bio-informatics tool for biomarker assessment and outcome-based cut-point optimization, Clinical cancer research : an official journal of the American Association for Cancer Research, 10 (2004) 7252-7259.

[5] R. Falotico, P. Quatto, Fleiss’ kappa statistic without paradoxes, Quality & Quantity, 49 (2015) 463-470.

[6] M. Fitzgerald, B.R. Saville, R.J. Lewis, Decision curve analysis, JAMA, 313 (2015) 409-410.

[7] A.J. Vickers, E.B. Elkin, Decision curve analysis: a novel method for evaluating prediction models, Med Decis Making, 26 (2006) 565-574.

[8] A. Zaitsev, M. Chelushkin, D. Dyikanov, I. Cheremushkin, B. Shpak, K. Nomie, V. Zyrin, E. Nuzhdina, Y. Lozinsky, A. Zotova, S. Degryse, N. Kotlov, A. Baisangurov, V. Shatsky, D. Afenteva, A. Kuznetsov, S.R. Paul, D.L. Davies, P.M. Reeves, M. Lanuti, M.F. Goldberg, C. Tazearslan, M. Chasse, I. Wang, M. Abdou, S.M. Aslanian, S. Andrewes, J.J. Hsieh, A. Ramachandran, Y. Lyu, I. Galkin, V. Svekolkin, L. Cerchietti, M.C. Poznansky, R. Ataullakhanov, N. Fowler, A. Bagaev, Precise reconstruction of the TME using bulk RNA-seq and a machine learning algorithm trained on artificial transcriptomes, Cancer cell, 40 (2022).

[9] J. Liu, H. Chen, G. Qiao, J.-T. Zhang, S. Zhang, C. Zhu, Y. Chen, J. Tang, W. Li, S. Wang, H. Tian, Z. Chen, D. Ma, J. Tian, Y.-L. Wu, PLEK2 and IFI6, representing mesenchymal and immune-suppressive microenvironment, predicts resistance to neoadjuvant immunotherapy in esophageal squamous cell carcinoma, Cancer Immunol Immunother, 72 (2023) 881-893.
